# Dynamic terahertz spectroscopy of gas molecules mixed with unwanted aerosol under atmospheric pressure using fibre-based asynchronous-optical-sampling terahertz time-domain spectroscopy

**DOI:** 10.1038/srep28114

**Published:** 2016-06-15

**Authors:** Yi-Da Hsieh, Shota Nakamura, Dahi Ghareab Abdelsalam, Takeo Minamikawa, Yasuhiro Mizutani, Hirotsugu Yamamoto, Tetsuo Iwata, Francis Hindle, Takeshi Yasui

**Affiliations:** 1 Graduate School of Science and Technology, Tokushima University, 2-1, Minami-Josanjima, Tokushima 770-8506, Japan; 2 JST, ERATO, MINOSHIMA Intelligent Optical Synthesizer Project, 2-1, Minami-Josanjima, Tokushima 770-8506, Japan; 3 Graduate School of Engineering, Osaka University, 2-1, Yamadaoka, Suita, Osaka 565-0871, Japan; 4 Center for Optical Research and Education, Utsunomiya University, 7-1-2, Yoto, Utsunomiya, Tochigi 321-858, Japan; 5 Laboratoire de Physico-Chimie de l’Atmosphère, Université du Littoral Côte d’Opale, 189A Av. Maurice Schumann, Dunkerque 59140, France

## Abstract

Terahertz (THz) spectroscopy is a promising method for analysing polar gas molecules mixed with unwanted aerosols due to its ability to obtain spectral fingerprints of rotational transition and immunity to aerosol scattering. In this article, dynamic THz spectroscopy of acetonitrile (CH_3_CN) gas was performed in the presence of smoke under the atmospheric pressure using a fibre-based, asynchronous-optical-sampling THz time-domain spectrometer. To match THz spectral signatures of gas molecules at atmospheric pressure, the spectral resolution was optimized to 1 GHz with a measurement rate of 1 Hz. The spectral overlapping of closely packed absorption lines significantly boosted the detection limit to 200 ppm when considering all the spectral contributions of the numerous absorption lines from 0.2 THz to 1 THz. Temporal changes of the CH_3_CN gas concentration were monitored under the smoky condition at the atmospheric pressure during volatilization of CH_3_CN droplets and the following diffusion of the volatilized CH_3_CN gas without the influence of scattering or absorption by the smoke. This system will be a powerful tool for real-time monitoring of target gases in practical applications of gas analysis in the atmospheric pressure, such as combustion processes or fire accident.

One interesting application of gas analysis is in monitoring of gas molecules mixed with unwanted aerosols, such as smoke, soot, dust, mist, fog, haze, fume, and so on. For example, dynamic analysis of combustion gas is required to realize the efficiency of combustion process from viewpoints of global environment and saving energy. On the other hand, real-time sensing of hazardous, toxic, or flammable gas in the smoke is important to avoid the secondary disaster in the fire accident. Against this backdrop, there is a strong demand for methods that allow dynamic analysis of molecular gases without the influence of unwanted aerosols. Although the target gas in those applications has relatively high concentration under atmospheric pressure (typically, a few hundreds ppm to a few tens %), the aerosols that are mixed with the collected samples makes the gas analysis difficult. For example, although gas chromatography^1^ has high sensitivity, the measurement is not real time, and it has many limitations due to the capture technology of unwanted aerosols used in sample preprocessing. On the other hand, infrared absorption spectroscopy^2^ is rapid, but the influence of light scattering by aerosols reduces the analysis performance. Therefore, a technology that allows the target gas in aerosols to be analyzed quickly and with high precision without the need for sample preprocessing is highly desirable.

The THz region (frequency range 0.1–10 THz, wavelength range 30–3000 μm) is a characteristic frequency band in which many absorption lines due to rotational transitions of polar gas molecules appear^3^,^4^. Instead of the intermolecular vibration spectrum observed in the infrared region, if the molecular rotational transition spectrum observed in the THz region could be used, high selectivity and high sensitivity would be expected. In addition, from the relationship between the wavelength of THz radiation and the size of minute particles, there is less susceptibility to optical scattering by aerosols^5^. Therefore, under conditions in which aerosols are mixed with the gas to be analyzed, as in the combustion processes or the fire accident, THz spectroscopy is considered to be a useful technique for simultaneously analyzing the target gas molecule species in a straightforward and rapid manner.

In order to discriminate the target gas at atmospheric pressure, a spectroscopic technique employed must have a spectral resolution matched to the atmospherically broadened absorption lines (typically, a few to a few tens GHz), reasonable spectral accuracy and a broad spectral coverage in the THz region. Typical examples of spectroscopic techniques in the THz region include THz frequency-domain spectroscopy (THz-FDS) with coherent tunable continuous-wave THz (CW-THz) radiation, THz time-domain spectroscopy (THz-TDS) with coherent broadband THz radiation, and Fourier transform far-infrared spectroscopy (FT-FIR) with incoherent broadband THz radiation.

In THz-FDS, a spectrum is obtained by frequency scanning narrow-band CW-THz radiation. The frequency-multiplied microwave sources (FMMS) provide fast sweeping of high spectral purity without mode hopping, high brightness, and exact frequency determination with electronic frequency reference to THz-FDS; however, the tuning range of a single FMMS is usually limited within 10 to 20% of a center frequency^6^,^7^. The THz-FDS with backward wave oscillator (BWO), also called to fast-scan submillimeter spectroscopy technique (FASSST), can extend the frequency coverage more widely while maintaining the similar resolution^8^. However, in these THz-FDSs, it is still difficult to fully cover the frequency range from 0.1 THz to a few THz at once; it is necessary to select a suitable FMMS or BWO tube depending on the frequency band used, due to the limited tuning range. More importantly, these THz-FDSs are not always ideal for gas molecules in atmospheric pressure due to mismatching of the narrow CW-THz linewidth and the atmospherically broadened absorption lines although they are highly promising for high-resolution spectroscopy of gas molecules in low pressure. Photomixing of two tunable near-infrared lasers is a promising approach to achieving broader continuous tuning while maintaining moderate spectral resolution in THz-FDS^9^; however, the accessible spectral range by the continuous tuning is usually narrower than that of THz-TDS and FT-FIR due to the mode hopping of the near-infrared lasers. For example, a tunable CW-THz signal generator (frequency range = 200 to 500 GHz, tuning speed = 375 GHz/s, and frequency resolution = 500 MHz) has been applied to active gas sensing^10^. Also, a tunable CW-THz signal generator phase-locked to an optical frequency comb has been used for the analysis of formaldehyde with sub-MHz uncertainty^11^. However, the need for a cryogenically cooled receiver and the limitation of the continuous tuning range may hinder the wide adoption of these methods.

On the other hand, with THz-TDS^12^ and FT-FIR^13^, in which a spectrum is obtained by Fourier transforming the temporal waveform or interferogram of the broadband THz radiation, it is possible to obtain a wideband spectrum (1 THz or more) at one time. For example, real-time sensing of polar molecular gases has been achieved by THz-TDS^14^. However, since frequency scaling of the spectrum is performed based on the amount of movement of a mechanical stage, the spectral accuracy is relatively low. Use of the mechanical time-delay scanning also hinders acquisition of a high-resolution spectrum in real time. Thus, conventional THz spectroscopy has advantages and disadvantages, and there have been little attempts to apply them to the real-time analysis of the target gas mixed with aerosols under atmospheric pressure.

Since the limits on spectral resolution, accuracy, and measurement time in the conventional THz-TDS are due to the mechanical time-delay scanning, THz-TDS without the need for mechanical scanning should be able to resolve these problems. One promising method is asynchronous-optical-sampling THz-TDS (ASOPS-THz-TDS) using two mode-locked femtosecond lasers with slightly mismatched repetition frequencies^15^,^16^,^17^,^18^. Since ASOPS-THz-TDS enables us to expand the picosecond time scale of a THz pulse by a temporal magnification factor (*TMF*) based on the principle of ASOPS, a standard oscilloscope or data acquisition board can directly measure the slowed-down ASOPS signal in the RF region. The non-mechanical feature of time-delay scanning in this method provides three attractive features for practical gas analysis with THz-TDS. First, since the measurement time window can be arbitrarily selected within one repetition period, the spectral resolution can be optimized to fit THz spectral signatures of gas molecules at atmospheric pressure. Use of the suitable spectral resolution compensates low brightness of the broadband THz radiation and maintains moderate signal-to-noise ratio (SNR). Second, the data acquisition time can be reduced to less than one second while maintaining moderate spectral resolution. Third, an optical configuration that does not need free-space optics and mechanical time-delay scanning of laser beams can be constructed based on dual fibre lasers and fibre-coupled photoconductive antennas (PCAs)^19^, providing attractive features for practical use, such as compactness, robustness, flexibility, and an alignment-free configuration. Furthermore, phase locking of the repetition frequencies of the dual femtosecond lasers to a microwave or radio-frequency (RF) frequency standard guarantees high spectral accuracy in the THz spectrum.

In this article, we applied a fibre-based ASOPS-THz-TDS system to the dynamic THz spectroscopy of acetonitrile (CH_3_CN) gas, which is a target gas, in the presence of smoke, which is an unnecessary aerosol, at the atmospheric pressure.

## Results

### Experimental setup

**Figure 1:**
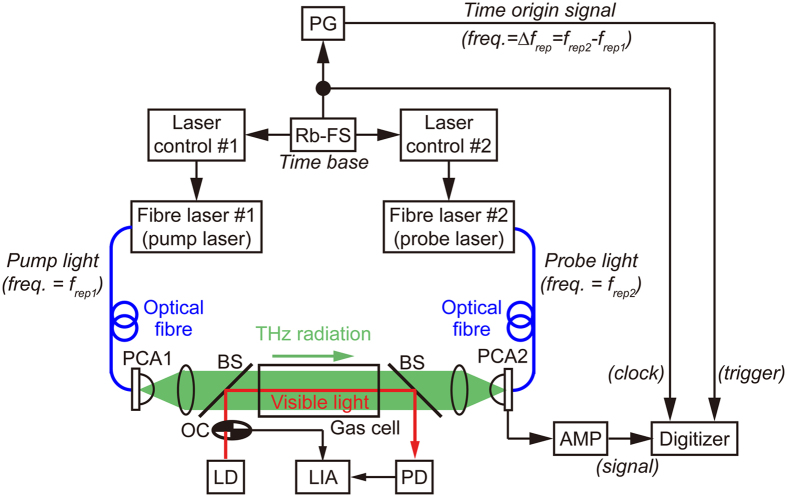
Experimental setup. Rb-FS: rubidium frequency standard; PCA1: strip-line-shaped LT-InGaAs/InAlAs photoconductive antenna for THz generation; BSs: pellicle beam-splitters; PCA2: dipole-shaped LT-InGaAs/InAlAs photoconductive antenna for THz detection; AMP: current preamplifier; LD: laser diode (λ = 635 nm); OC: optical chopper; PD: photodetector; LIA: lock-in amplifier; PG: pulse generator.

Figure 1 shows a schematic diagram of the experimental setup, which contains dual repetition-frequency-stabilized, mode-locked Er-fibre lasers (center wavelength = 1550 nm, pulse duration = 50 fs, *f*_*rep1*_ = 250,000,000 Hz, *f*_*rep2*_ = 250,000,050 Hz, *∆f*_*rep*_ = *f*_*rep2*_  −  *f*_*rep1*_ = 50 Hz), a pair of fibre-coupled LT-InGaAs/InAlAs PCAs, and a THz optical setup including a gas cell. The pulsed THz radiation was emitted from a strip-line-shaped LT-InGaAs/InAlAs PCA emitter (PCA1) triggered by pump light, passed through the gas cell after being collimated with a THz lens, and was then focused onto a dipole-shaped LT-InGaAs/InAlAs PCA detector (PCA2) triggered by probe light. The temporal waveform of the pulsed THz electric field was obtained at 50 Hz with a sampling interval of 100 fs by acquiring the current signal from PCA2 with a digitizer. After accumulating 50 temporal waveforms, we selected the first quarter of its whole temporal waveform in the pulsed THz electric field (pulse period = 4 ns), corresponding to a time delay from 0 to 1 ns, to obtain a THz amplitude spectrum by Fourier transform. Finally, a THz power spectrum with a frequency resolution of 1 GHz was acquired at a scan rate of 1 Hz for dynamic THz spectroscopy of the sample gas.

### Immunity of THz radiation to scattering by aerosols

**Figure 2:**
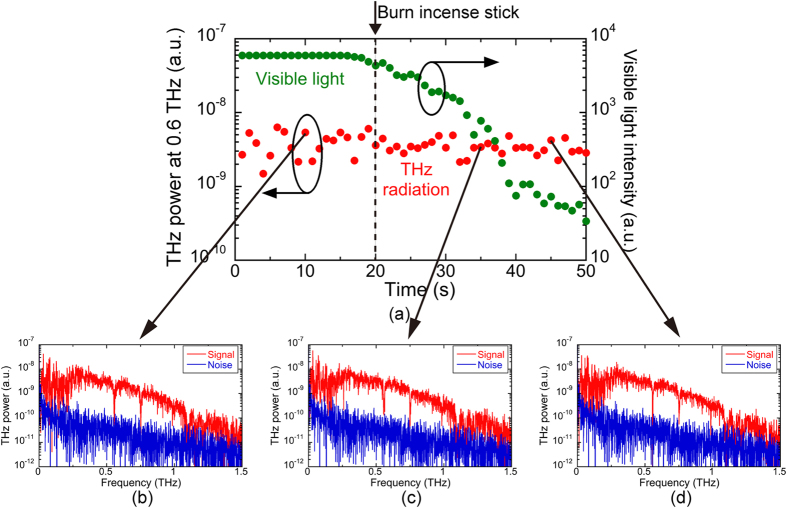
(**a**) Temporal change of THz power at 0.6 THz (red plot, left vertical axis) and visible light intensity at 635 nm (green plot, right vertical axis) before and after injection of smoke by burning an incense stick. THz power spectra (**b**) before (=10 s), (**c**) during (=35 s), and after (=45 s) burning the incense stick.

To experimentally confirm the robustness of THz radiation to scattering by the aerosols, we measured the signal attenuation of broadband THz radiation and visible light in the presence of smoke from the incense stick. The THz radiation and visible light from a laser diode (LD; wavelength = 635 nm, average power = 3 mW) were made to collinearly overlap with a pellicle beam-splitter (BS) and then co-propagated in the gas cell A (length = 200 mm, diameter = 50 mm) used for a sample gas mixed with aerosol in the atmospheric pressure (see Fig. 1). After passing through the cell, the visible light was separated again by another BS and was measured using a combination of an optical chopper (OC), a photodetector (PD), and a lock-in amplifier (LIA). The THz power spectrum and the visible light intensity were measured at intervals of 1 s during a 50 s period. The incense stick was burned for 20 s after the start of the measurement in the gas cell A. Red and green plots in Fig. 2(a) show the temporal change of the THz power at 0.6 THz and the visible light intensity before and after burning the incense stick. Although the scattering by the smoke reduced the visible light intensity by about 1/175, the THz power was almost constant before and after burning the incense stick. THz power values (arbitrary unit) were (3.97 × 10^−09^) ± (1.44 × 10^−09^) [(mean) ± (standard deviation)] before burning, and (3.44 × 10^−09^) ± (7.57 × 10^−10^) after burning. THz power spectra of the signal and noise before, during, and after burning the incense stick are shown in Fig. 2(b–d). Although sharp absorption lines always appeared at 0.557 THz and 0.752 THz due to the atmospheric water vapor inside and outside of the cell, the spectral shape was not distorted at all during the measurement, indicating no scattering or absorption by the smoke. In this way, we confirmed the high robustness of THz radiation to the smoke from the viewpoint of both the THz power value and the spectral shape. Such the robustness of THz radiation to the smoke is in good agreement with that to the dust clouds in the previous research^5^.

### Static THz spectroscopy of CH_3_CN gas

**Figure 3:**
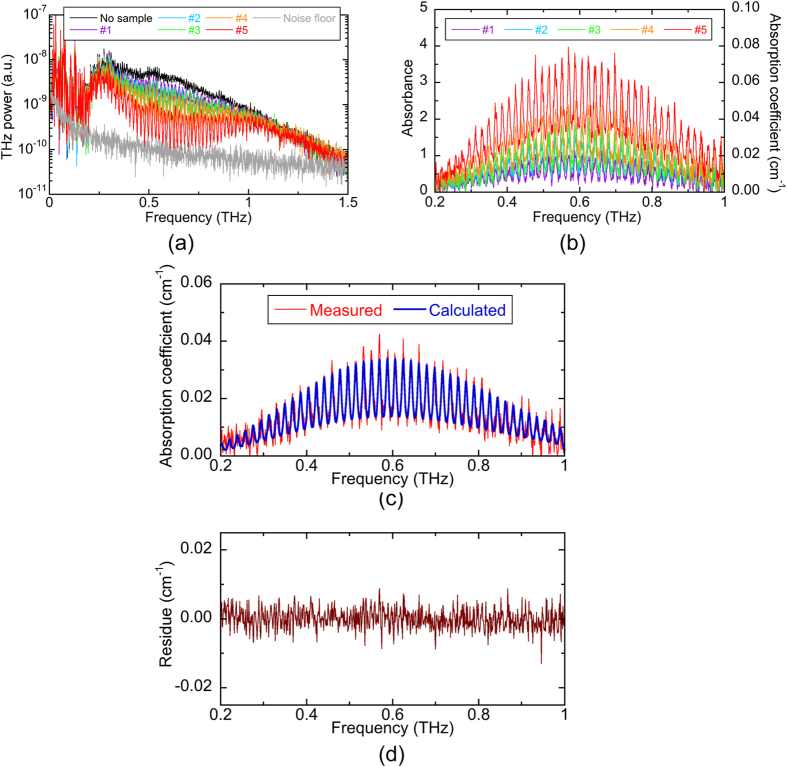
(**a**) Change of THz power spectra with respect to different concentrations of CH_3_CN gas. (**b**) Change of absorbance spectrum and the corresponding absorption coefficient spectrum with respect to different concentration. (**c**) Comparison of the measured absorption coefficient spectrum and the calculated one for the gas sample #3 and (**d**) residue between them for the gas sample #3.

We next measured the absorption spectra of CH_3_CN gas with different concentrations at static condition. In the gas cell B (length = 500 mm, diameter = 40 mm), CH_3_CN gas was diluted with nitrogen (N_2_) gas to change the CH_3_CN gas concentration without interference due to THz absorption by other gases, such as water vapor. We prepared 5 samples of CH_3_CN gas with different concentrations (sample #1, #2, #3, #4, and #5) by changing the ratio of the partial pressure between CH_3_CN gas and N_2_ gas, while keeping the total pressure of the gas mixture fixed at the atmospheric pressure (=101.3 kPa). The gas concentration of each sample was unknown. We repeated the THz power spectrum measurements 10 times at time intervals of 1 s, and then obtained its mean for each concentration, which is corresponding to a measurement rate of 0.1 Hz. Figure 3(a) shows the comparison of the mean THz power spectrum for 5 gas samples. For reference, the power spectra of no samples and the noise signal (the noise floor) are also shown in the same graph. Many manifolds of absorption lines were superimposed more deeply on the broad THz spectrum depending on the gas samples, indicating that the gas concentration was increased. Figure 3(b) shows the absorbance spectrum (left vertical axis) and the corresponding absorption coefficient spectrum (right vertical axis) with respect to different gas samples. The observed *J* manifolds regularly spaced by 18.4 GHz were in good agreement with the spectral features by a rotational constant *B* (=9.199 GHz) of CH_3_CN^20^. Spectral overlapping of adjacent *J* manifolds implies that pressure broadening was achieved to several GHz at atmospheric pressure. Difference of the absorption spectra among them indicated that each gas sample had different gas concentration from each other.

We next applied the fitting model (see Methods) to five spectra in Fig. 3(b) to determine the gas concentration for each sample. Figure 3(c) shows the comparison of the measured spectrum (red line) and the calculated one (blue line) for the gas sample #3. Residue between them is given in Fig. 3(d). Such good fitting result indicated that the proposed fitting model was suitable for the quantification of CH_3_CN gas in the atmospheric pressure. We also obtained the similar fitting result for other samples (not shown). Table 1 summarizes the gas concentration by volume, the estimated detection limit, the linewidth *Δv*, the linewidth *Δv* uncertainty, and root mean square (RMS) of the residue for 5 gas samples. The detection limit and the linewidth uncertainty are based on the confidence interval determined during the fitting procedure. The gas concentration was determined to be 0.341% for #1, 0.497% for #2, 0.580% for #3, 0.800% for #4, and 1.356% for #5. On the other hand, the mean and standard deviation of the detection limit was 36 ± 11 ppm.

### Dynamic THz spectroscopy of CH_3_CN gas mixed with smoke

Finally, we performed dynamic THz spectroscopy of CH_3_CN gas mixed with smoke in the gas cell A at the atmospheric pressure. The enclosed gas cell was filled with smoke beforehand by placing the burning incense stick into the gas cell via the side window. A few droplets of CH_3_CN liquid were dripped in the gas cell from its side window after opening it for a while, and then the window was closed again. The change in the CH_3_CN gas concentration, associated with volatilization of CH_3_CN droplets and the following diffusion of the volatilized CH_3_CN gas, was monitored under smoky conditions by dynamic THz spectroscopy. After putting the CH_3_CN droplets in the gas cell, we recorded the power spectrum of the broad THz radiation with a spectral resolution of 1 GHz every 1 s for 50 s.

**Figure 4:**
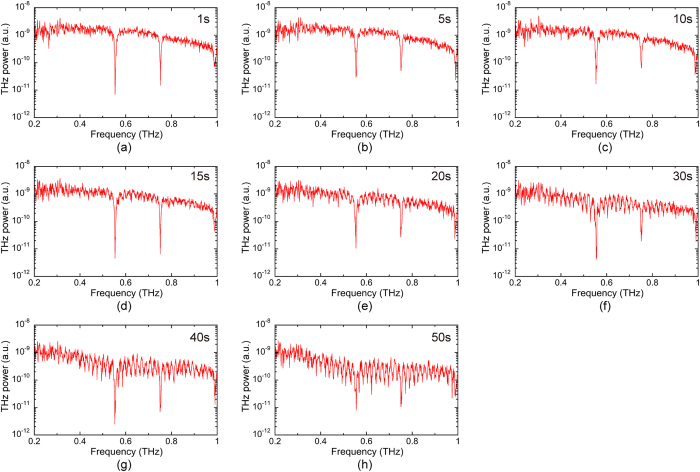
Temporal change of THz power spectra during volatilization of CH_3_CN droplets and diffusion of CH_3_CN gas in the gas cell A filled with smoke (see Video 1). (**a**) 1 s, (**b**) 5 s, (**c**) 10 s, (**d**) 15 s, (**e**) 20 s, (**f**) 30 s, (**g**) 40 s, and (**h**) 50 s after volatilization of the CH_3_CN droplets.

Figure 4 and Video 1 show the temporal change of the THz power spectrum with respect to elapsed time. Immediately after the start of measurement, only the absorption lines of atmospheric water vapor are visible in the power spectrum [see Fig. 4(a)]. During the first 10 seconds, no absorption lines of CH_3_CN gas can be clearly identified because the gas concentration was below the lower detection limit in the present system [see Fig. 4(b,c)]. The absorption lines of CH_3_CH can be observed at 15 s [see Fig. 4(d,e)] and can be easily distinguished after 30 s [see Fig. 4(f–h)]. In this way, we succeeded in monitoring the temporal change of absorption lines superimposed on the broad THz spectrum in real time.

**Figure 5:**
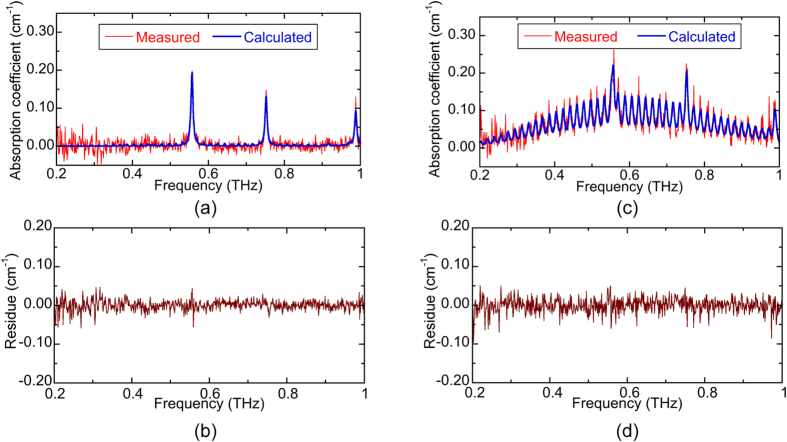
(**a**) Comparison of the measured absorption and calculated spectra along with the residue (**b**) between them for the CH_3_CH gas mixed with smoke at t = 5 s. (**c**) Comparison of the measured absorption and calculated spectra at t = 50 s with the residue (**d**).

A similar fitting procedure was applied to the spectra after being modified to account for the contributions of both CH_3_CN and water vapor (see Methods). Figure 5(a) shows the comparison of the measured spectra (red line) and the calculated one (blue line) at t = 5 s, while Fig. 5(b) indicates the residue between them. At this time, only three rotational transitions of the water vapor are visible, indicating that the water absorption was dominant. The calculated absorption spectrum is in good agreement with the measured data. Figure 5(c) compares the measured spectra (red line) and the calculated one (blue line) at t = 50 s, while Fig. 5(d) indicates the residue between them. In this case, both contributions of CH_3_CN and water vapor are observed in the measured absorption spectrum, they are also correctly reproduced in the calculated spectrum.

**Figure 6:**
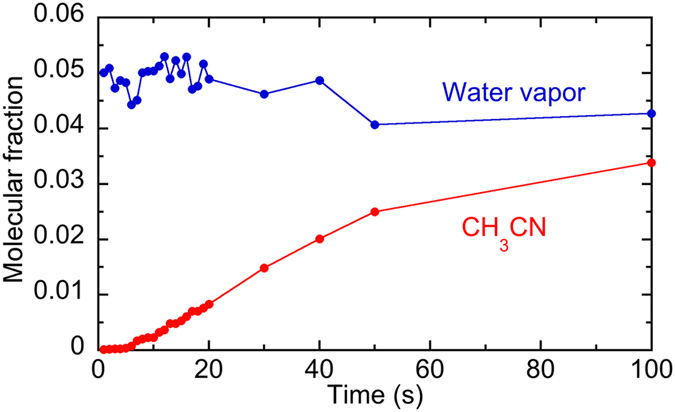
Temporal change of the molecular fraction in CH_3_CN and water vapour when CH_3_CN droplets were volatilized and the volatilized CH_3_CN gas was diffused in the gas cell filled with the smoke.

The molecular fraction of CH_3_CN and water vapor was determined as a function of time and shown in Fig. 6. As the evaporation of the liquid CH_3_CN increases we observed a slight reduction in the water concentration due to its displacement. The CH_3_CN concentration determined at t = 100 s is probably underestimated due to the saturation of the spectrometer. The uncertainty of CH_3_CN concentration is 200 ppm that can be considered to be the detection limit of the present system for a measurement time of 1 s. The difference of the detection limit between the static THz spectroscopy (=36 ppm, see Fig. 3) and the dynamic THz spectroscopy (=200 ppm, see Fig. 4) is mainly due to the difference of the effective data acquisition time between them rather than the existence of the smoke. An identical procedure for water vapor gave an estimated detection limit of 0.1%.

## Discussion

We demonstrated the dynamic THz spectroscopy of CH_3_CN gas mixed with smoke under the atmospheric pressure. While the CH_3_CN gas is the favorable gas for the THz spectroscopy in the atmospheric pressure due to its strong absorption and spectral structure, it is related with incomplete combustion of nylon fabric^10^. For practical applications of the present system, an estimation of its sensitivity for alternative gases is of great interest.

The hydrocarbon (HC) combustion is the primary process in the burning of fossil fuel, and the complete combustion of any hydrocarbon in sufficient oxygen produces carbon dioxide (CO_2_) and water. Although CO_2_ is a good indicator of the combustion process due to being a polar molecule, its absorption is very weak in THz region. On the other hand, incomplete combustion produces additional gases, such as carbon monoxide (CO), HC gas, and nitrogen oxide (NO_x_), some of which are polar molecules and hence display THz spectral signatures. Therefore, THz spectroscopy of these gases will be useful to investigate combustion efficiency. In the case of the fire accident, harmful gases, such as CO, CO_2_, hydrogen cyanide (HCN), hydrogen chloride (HCl), sulfur oxide (SO_x_), and NO_x_, are generated depending on the nature and quantity of combustibles, the elapsed time from the start of fire, and the distance from the seat of the fire. Since some of these gases are also polar molecules, THz spectroscopy can be used for monitoring of these gases^10^.

The 200 ppm CH_3_CN detection limit was achieved at atmospheric pressure of smoky condition because the molecule’s spectral signature contains many strong transitions in the band from 200 GHz to 1 THz. Accounting for the contribution of all the overlapping pressure broadened lines provided the required sensitivity which can not be obtained by monitoring a single isolated line or a smaller group of overlapping lines. Table 2 shows a comparison of the number of absorption lines, total integrated absorption intensity, and estimated detection limit between CH_3_CN gas and other gases related with combustion process and fire accident, calculated over the same spectral range from 200 GHz to 1 THz^4^. Although the influence of pressure broadening is not considered there should be a linear relationship between the total integrated absorption intensity and the detection limit assuming similar pressure broadening for these gases. The detection limit of each gas is estimated based on the total integrated absorption and the detection limit obtained for the measured data of CH_3_CN. Although the sensitivity of the present system to CO and NO_2_ is somewhat limited the identification and quantification of multiple species such as HCN, HCl, SO, and SO_2_ has potential. Indeed the present demonstration showed how a relatively simple fitting procedure was able to simultaneously quantify two different gases. Optimization of the data processing procedure will be critical in order to increase the number of molecules which may be considered simultaneously. The extension of the spectral range undergoing analysis will also assist in the differentiation between the species. Nevertheless the matching of the instrument spectral resolution to the expected molecular linewidth leads to an optimal solution in the compromise between resolution and acquisition time.

## Conclusions

We applied a fibre-based ASOPS-THz-TDS system with a spectral resolution of 1 GHz to the dynamic THz spectroscopy of CH_3_CN gas mixed with smoke at the atmospheric pressure. Without the influence of scattering or absorption by the smoke, many *J* manifolds of rotational transitions in CH_3_CN molecules were spectrally resolved at atmospheric pressure at a measurement rate of 1 Hz. Using the fitting model and considering all the spectral contributions of the numerous closely packed *K* components in each of *J* manifolds from 0.2 THz to 1 THz, the temporal change of CH_3_CN gas concentration was monitored with an uncertainty of 200 ppm. The multiplex nature of this spectrometer also allowed the water concentration to be determined simultaneously with an uncertainty of 0.1%. We confirmed that the CH_3_CN concentrations increased from zero to 3.4% during the volatilization of CH_3_CN droplets, and that the volatilized CH_3_CN gas diffused in the gas cell filled with smoke while the water vapour concentration fell slightly from 0.050 to 0.043. Also, we estimated the lower detection limit of several gases related with the combustion process and the fire accident from comparison of the integrated absorption intensity, and show a potential of the present system for monitoring of HCN, HCl, SO, and SO_2_.

Although the further enhancement of SNR and dynamic range (DR) is required for more precise analysis of the target gas with a lower concentration or absorption, the features of our fibre-based ASOPS-THz-TDS system, namely, compactness, robustness, flexibility, and an alignment-free configuration, will be useful for open-field gas analysis in the combustion process or fire accident. Also, use of unstabilized dual femtosecond lasers in the adaptive sampling technique^21^ will increase the versatility of this fibre-based ASOPS-THz-TDS system in practical gas analysis.

## Methods

### Sample gas and aerosol

We selected acetonitrile (CH_3_CN) as a sample gas mixed with aerosol because of its strong absorption and characteristic spectral structure in gas phase. Since CH_3_CN has a symmetric top molecular structure with a rotational constant*, B*, of 9.199 GHz, and a centrifugal distortion constant, *D*_*JK*_, of 17.74 MHz^20^, the frequencies of rotational transitions are given by


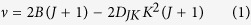


where *J* and *K* are rotational quantum numbers. From this equation, the molecule displays two characteristic features in its THz spectrum. The first term in Eq. (1) indicates that *J* manifolds of absorption lines regularly spaced by *2B* (=18.398 GHz) appear. The second term indicates that each manifold includes a series of closely spaced absorption lines of decreasing strength, namely *K* components, due to *2D*_*JK*_. In low pressure, CH_3_CN gas indicates the characteristic spectral structures with both GHz and MHz orders, as mentioned by *J* manifolds and *K* components. On the other hand, in the atmospheric pressure, many closely packed *K* components in each of *J* manifolds overlap to each other due to the pressure broadening. The resulting signal, namely unresolved *K* components, is significantly boosted compared with the intensity of a single transition. In this case, the extra strength approaches a factor of ten. This is one advantage of atmospheric-pressure spectroscopy of symmetric top molecules. If a spectral resolution of around 1 GHz is achieved, only *J* manifolds of rotational transitions regularly spaced by *2B* will be observed at atmospheric pressure.

Regarding aerosol, we used dense smoke from an incense stick to mix with CH_3_CN. In this smoke, visible light is largely attenuated by scattering, whereas THz radiation exhibits little scattering due to the relation between the particle diameter and the wavelength.

### Detailed experimental setup

Figure 1 shows a schematic diagram of the experimental setup. The fibre-based ASOPS-THz-TDS system consists of dual mode-locked Er-fibre lasers (ASOPS TWIN 250, Menlo Systems, center wavelength = 1550 nm, pulse duration = 50 fs), a pair of fibre-coupled LT-InGaAs/InAlAs PCAs (TERA 15-TX-FC and TERA 15-RX-FC, Menlo Systems), and a THz optical setup including a gas cell. The individual mode-locked frequencies of the fibre lasers (*f*_*rep1*_ = 250,000,000 Hz and *f*_*rep2*_ = 250,000,050 Hz) were stabilized by two independent laser control systems (RMS timing jitter <150 fs in the range 0.1 Hz–500 kHz) referenced to a rubidium frequency standard (Rb-FS; FS725, Stanford Research Systems, accuracy = 5 × 10^−11^ and instability = 2 × 10^−11^ at 1 s). Thus, the frequency difference between them (*∆f*_*rep*_ = *f*_*rep2*_  −  *f*_*rep1*_ = 50 Hz) was also stabilized. The pulsed THz radiation was emitted from a strip-line-shaped LT-InGaAs/InAlAs PCA emitter (PCA1; bias voltage = 20 V) triggered by pump light (mean power = 20 mW), passed through the gas cell after being collimated with a THz lens (Tsurupica, Pax Co., focal length = 50 mm, diameter = 50 mm), and was then focused onto a dipole-shaped LT-InGaAs/InAlAs PCA detector (PCA2) triggered by probe light (mean power = 9 mW). The current signal from PCA2 was amplified and converted into a voltage signal with a current preamplifier (AMP; bandwidth = 1 MHz, gain = 4 × 10^6^ V/A). Since the temporal waveform of the current signal is corresponding to that of the temporally magnified THz pulse signal (repetition period = 1/*∆f*_*rep*_ = 20 ms), the current signal was acquired within a time window size of 20 ms with a digitizer (sampling rate = 2 × 10^6^ samples/s, number of sampling points = 40,000, resolution = 20 bit) by using an RF pulse signal (pulse duration = 1 μs, frequency = *∆f*_*rep*_ = 50 Hz) as a trigger signal, which was synthesized from a pulse generator (PG) phase-locked to Rb-FS. Since this pulse signal was in synchronization with *∆f*_*rep*_ due to use of the common time-base from Rb-FS, it was equivalent to an optical trigger signal used in the previous ASOPS-THz-TDS systems^15^,^16^,^17^,^18^. Such an electric trigger generator enables an all-fibre-based ASOPS-THz-TDS system, except for the THz optical setup. The configuration of the digitizer with a *TMF* of 5,000,000 (=*f*_*rep1*_/*∆f*_*rep*_) is equivalent to the acquisition of a single THz pulse (repetition period = 1/*f*_*rep1*_ = 4 ns), corresponding to a sampling interval of 100 fs with a time window of 4 ns. We acquired the temporal waveform of the THz pulse at a scan rate of 50 Hz and accumulated 50 temporal waveforms to increase SNR and DR. To optimize the spectral resolution for THz gas spectroscopy at atmospheric pressure, we selected the first quarter of the whole temporal waveform for the pulsed THz radiation, corresponding to a time delay from 0 to 1 ns, and calculated its Fourier transform to obtain a THz amplitude spectrum. Finally, a THz power spectrum with a frequency resolution of 1 GHz was acquired at a scan rate of 1 Hz for dynamic THz spectroscopy of the sample gas.

We prepared two kinds of gas cells, gas cell A used for a sample gas mixed with aerosol and gas cell B used for a sample gas diluted with buffer gas. The gas cell A was made of a cylindrical enclosure (length = 200 mm, diameter = 50 mm) and two parallel optical windows (Tsurupica, Pax Co., thickness = 10 mm, diameter = 50 mm), and was used for a sample gas mixed with aerosols at atmospheric pressure. This gas cell was not so tightly sealed to maintain the atmospheric pressure and provide the sufficient oxygen when burning the incense stick. To introduce the sample and the aerosols into the gas cell A, a small window in the side wall of the cylindrical enclosure was used. On the other hand, the gas cell B is tightly sealed (length = 500 mm, diameter = 40 mm, window material = white polyethylene, pressure range = 0.2 Pa to 101.3 kPa, pressure leakage = 0.98 Pa/h), and was used for the diluted sample gas without an aerosol.

### Quantification of gas concentration

The concentration of the absorbing gas can be quantified by determining the line area of a single absorption spectrum^22^. The line area in the atmospheric pressure was obtained by fitting the measured absorbance spectrum α(ν)*L* to a Lorentzian profile as follows


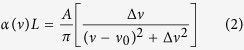


where *A* is the line area, *ν*_*0*_ is the center frequency, *∆ν* is the half width at half maximum (HWHM). Then, the gas concentration *N* (molecules/cm^3^) can be determined from the following equation


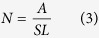


where *S* is the line intensity and *L* is the interaction length.

However, in the case of THz spectroscopy of CH_3_CN gas in atmospheric pressure, spectral overlapping of many closely packed *K* components complicates the direct interpretation of the data. A better approach would be to use all the spectral contribution together (about 400 lines from 0.2 THz to 1 THz, for example) rather than a single spectral contribution. Since tabulated values for *ν*_*0*_ and *S* of individual *K* components in CH_3_CN can be found in JPL Molecular Spectroscopy catalog^4^, we can construct the overlapped spectrum as a line diagram. If we apply a pressure broadening to the line diagram, we should be possible to obtain a spectrum that has the same shape as the measured profile. Once we have the shape adjusting the total integrated intensity (integrated from 0.2 THz to 1 THz), we will be able to fit the various spectra measured at the different concentration levels. We here assumed that influence of the pressure broadening is common to all lines. This model was applied to the measured spectra using a standard non-linear fitting technique yielding linewidth *∆ν* and gas concentration *N*.

In dynamic THz spectroscopy of CH_3_CN gas mixed with smoke, we further consider the contribution of atmospheric water vapor because three rotational transitions (1_10_ ← 1_01_ at 0.557 THz, 2_11_ ← 2_02_ at 0.752 THz, and 2_02_ ← 1_11_ at 0.988 THz) superimposes on the spectrum together with the rotational transitions of CH_3_CN. We simultaneously quantified the concentrations of both CH_3_CN gas and water vapor following the same procedure.

## Additional Information

**How to cite this article**: Hsieh, Y.-D. *et al*. Dynamic terahertz spectroscopy of gas molecules mixed with unwanted aerosol under atmospheric pressure using fibre-based asynchronous-optical-sampling terahertz time-domain spectroscopy. *Sci. Rep*. **6**, 28114; doi: 10.1038/srep28114 (2016).

## Supplementary Material

Supplementary Information

Supplementary Video S1

## Figures and Tables

**Table 1:** Quantification of CH_3_CN concentration for 5 gas samples at different concentrations using static THz spectroscopy.

Gas sample	Acetonitrile concentration by volume (%)	Estimated detection limit (ppm)	Linewidth ∆*ν* (cm^−1^)	Linewidth ∆*ν* uncertainty (cm^−1^)	RMS
#1	0.341	20	0.152	0.0022	0.0016
#2	0.497	20	0.150	0.0015	0.0015
#3	0.580	40	0.147	0.0017	0.0023
#4	0.800	40	0.157	0.0016	0.0025
#5	1.356	40	0.167	0.0011	0.0029

**Table 2:** Comparison of the number of absorption lines, total integrated absorption intensity, and estimated detection limit between CH_3_CN gas and other gases related with combustion process and fire accident.

Molecule	Number of absorption lines	Total integrated intensity (nm^2^MHz)	Estimated detection limit
CH_3_CN	900	7.5	200 ppm
CO	7	0.0082	18%
NO_2_	3220	0.045	3%
HCN	9	7.9	200 ppm
HCl	2	0.10	1.5%
SO	135	2.4	600 ppm
SO_2_	2397	2.0	700 ppm
