# Supplementary material for: Dynamic terahertz spectroscopy of gas molecules mixed with unwanted aerosol under atmospheric pressure using fibre-based asynchronous-optical-sampling terahertz time-domain spectroscopy
Source: Sci Rep. 2016 Jun 15;6:28114. doi: 10.1038/srep28114 (PMC4908425; doi:10.1038/srep28114)
Supplement: Supplementary Information [file srep28114-s1.pdf]

Supplementary information

Video Legend

Video 1. Temporal change of THz power spectra during volatilization of CH_3CN droplets and diffusion of CH_3CN in the gas cell A filled with smoke. (a) 1 s, (b) 5 s, (c) 10 s, (d) 15 s, (e) 20 s, (f) 30 s, (g) 40 s, and (h) 50 s after volatilization of the CH_3CN droplets.

Manuscript #: SREP-16-08075

Authors: Yi-Da Hsieh, Shota Nakamura, Dahi Ghareab Abdelsalam, Takeo Minamikawa, Yasuhiro Mizutani, Hirotsugu Yamamoto, Tetsuo Iwata, Francis Hindle, and Takeshi Yasui

Title: Dynamic terahertz spectroscopy of gas molecules mixed with unwanted aerosol under atmospheric pressure using fibber-based asynchronous-optical-sampling terahertz time-domain spectroscopy

Journal: Scientific Reports
